# A meta-analysis on the effects of IT capability toward agility and performance: New directions for information systems research

**DOI:** 10.1371/journal.pone.0268761

**Published:** 2022-10-27

**Authors:** Karl Werder, Janek Richter

**Affiliations:** Cologne Institute for Information Systems, University of Cologne, Cologne, Germany; Universita degli Studi di Perugia, ITALY

## Abstract

Information technology (IT) capability is an organizational capability that enables organizations to acquire, deploy, combine, and reconfigure IT resources. As such, it is often investigated in conjunction with organizational agility—an organization’s ability to sense and respond to changes—and organizational performance. Studies on IT capability distinguish between reactive and proactive IT capability and identify varying effects in relation to agility and performance. While reactive IT capability supports and enhances work processes, proactive IT capability supports and enhances business strategies. In the light of the mixed results of prior research, we conduct a meta-analytical investigation into the varying effects that reactive and proactive IT capability have on organizational agility and organizational performance. We identified 6.436 studies from multiple sources that we systematically reduced to include 72 empirical studies in our analysis. Contrary to previous results and widely held opinion, our meta-analysis neither finds support for differences in effect size between reactive (r_+_ = 0.39, k = 34, 95% Confidence Interval (CI) [0.34, 0.44]) and proactive IT capability (r_+_ = 0.38, k = 21, 95% CI [0.31, 0.45]) toward agility (z = 0.68, p = 0.25), nor from reactive IT capability (r_+_ = 0.31, k = 43, 95% CI [0.26, 0.37]) and proactive IT capability (r_+_ = 0.33, k = 25, 95% CI [0.27, 0.40]) toward performance (z = 1.11, p = 0.13). Given the importance of IT capability, we discuss possible explanations and propose four areas for future research: latency, sequence, configurational, and theoretical multiplicity of IT capability.

## Introduction

Information technology (IT) capability is an organization’s ability to acquire, deploy, combine, and reconfigure IT resources and has been a significant research area for information systems (IS) researchers. IT capability is an important driver of organizational agility [1, 2] and it affects organizational performance [3, 4] (referred to as ‘agility’ and ‘performance’ from here on without further specification for reasons of brevity). Successful organizations are characterized by clever IT investments serving as digital options that help in coping with unanticipated developments [5]. For example, successful organizations are able to leverage their IT processes to adapt in the face of unanticipated changes such as when the worldwide pandemic struck and organizations had to send large parts of the work force into lockdown and to digitally work from home [6]. Another example includes strategic shifts of organizations by leveraging their digital options to arrive at completely new business solutions for their customers (e.g., Amazon’s entry into cloud business [7]). Although the extant literature varies in their exact conceptualizations of IT capability, distinction between two different arguments of how positive outcomes in agility and performance are achieved are prevalent: On the one hand, IT capability supports and enhances work processes [8], for example, through IT infrastructure capability [9] or IS integration [10]. On the other hand, IT capability supports and enhances the business strategy [8], for example, through outside-in IT capabilities [11] or IT business partnership [12].

Prior literature on IT capability makes two important assumptions for a unified understanding and synthesis of IT capability: First, the literature assumes that IT capability is a higher level construct and that it can be deconstructed in a number of dichotomous ways to resemble support for work processes vs business strategies [8]. Second, the literature assumes that both IT capability arguments are equally important [9]. However, we find strong variation in effect sizes of reported correlations. For example, some studies report slightly negative effects for the IT capability-performance relationship (e.g., r = -0.02 in [13]), while other report a strong positive effect (e.g., r = 0.52 in [14]). These results warrant a closer examination of the relationship as they suggest possible confounding effects.

Based on these observations, we suggest the need to conceptually and empirically distinguish between two types of IT capability related to work processes and business strategy. We distinguish the two types as reactive and proactive IT capability. Reactive IT capability reflects the organization’s ability to acquire, deploy, combine, and reconfigure IT resources with a focus on supporting and enhancing work processes. As such, reactive IT capability assists organizations to achieve operational outcomes, such as cost-effectiveness, quality, productivity, and customer services [15]. Proactive IT capability is an organization’s ability to acquire, deploy, combine, and reconfigure IT resources with a focus on supporting and enhancing business strategies. Hence, proactive IT capability enhances an organization’s capability to implement radical changes to business processes and to drive IT innovation [7].

Against this backdrop, we follow an evidence-based approach and conduct a meta-analytical investigation [16] for the effects of reactive and proactive IT capability toward agility and performance. This approach allows us to resolve identified variations in effect sizes and possible confounding effects. Although the literature on the effects of IT capability is rich, results have not been integrated to enhance our understanding of the comparative differences between both types of IT capabilities. The current state of research demands such a comparative view to enhance our understanding of differences in agility and the performance implications of reactive and proactive IT capability. Our main argument is that organizations need to distinguish between reactive and proactive IT capability. While reactive IT capability help organizations to achieve cost-effectiveness, proactive IT capability facilitate innovation. We formulate the following research question:

What are the effects of reactive and proactive IT capability toward agility and performance?

Motivated by the above research question, this article adopts a meta-analytical approach investigate 72 empirical studies. We integrate and synthesize earlier empirical findings on the effects of proactive and reactive IT capability on agility and performance. Given the substantial number of previous empirical studies, an integrative view is needed to advance our understanding of this subject. In contrast to individual studies’ results and while analyzing a broad spectrum of samples, we find that the effects of proactive and reactive IT capability do not differ on agility and they do not differ on performance as well. We discuss this counter-intuitive result that we refer to as IT capability fallacy–that is a widely held misleading believe about IT capability–along potential reasons. Based on our discussion, we develop directions for future research.

We make two contributions. First, we contribute a synthesis of prior findings on the IT capability-agility and IT capability-performance relationship. While prior research has often investigated the effect of a unitary IT capability construct, we distinguish between reactive and proactive IT capability at the level of operationalization, providing a comparative view to resolve the question of the mixed results produced by previous studies. Second, we identify the need for IT capability research to take different directions in the future. Relying on a broad range of empirical studies, we cannot suggest that a view that distinguishes between reactive and proactive IT capability alone is sufficient. This is especially alarming when viewed in the light of our dominant research tradition and given the number of studies relied upon. Hence, we develop future research directions and suggest four areas for future research on IT capability, IT-enabled agility, and IT-enabled performance. We suggest that future research needs to investigate the latency and sequence of individual IT capability, and their configurational and theoretical multiplicity with related factors.

Following this introduction, we review the literature on IT capability and explanations for its effects toward agility and performance. Thereafter, we present our sample, data collection and analytical approach of our research method before presenting the results. Following a discussion of our results in which we explore four possible explanations, we develop a research agenda for future research on IT capability. At the end, we present important limitations and conclude the article with important implications.

## Reactive and proactive IT capability

IT has been put forward as an important antecedent of agility [9]. The notion of IT capability stems from the resource-based view [17], which attributes superior performance to organizations that possess the right organizational resources and capabilities [18]. Generally, organizational capabilities such as IT capability are derived from organizational resources such as IT resources. IT resources comprise tangible technological resources (e.g., IT infrastructure), intangible IT-enabled resources (e.g., customer orientation), as well as human-IT resources (e.g., managerial IT skills [18]). An organization’s ability to use IT resources to its advantage has been the subject of extensive debate within the IS literature [8, 19]. Focusing on the organization’s IT capability instead of only relying on its IT resources has been found to be more adequate in dynamic business environments [20]. IT capability is defined as an organization’s ability to acquire, deploy, combine, and reconfigure IT resources in support and enhancement of work processes and business strategies [8]. We conceptualize IT capability for our study as a hierarchical structure in which specialized organizational capabilities are integrated into broader organizational capabilities [21]. Various conceptualizations of how specialized IT capabilities constitute organization-wide IT capability have been suggested by prior research [22, 23].

We follow the widely used seminal work of Sambamurthy and Zmud [8] that present IT capability’s support and enhancement of (1) work processes and (2) business strategies. The literature suggests that IT capability can be deconstructed in a number of dichotomous ways to resemble support for work processes vs business strategies–for example, inside-out vs. outside-in IT capabilities [11], IT exploitation vs exploration [24], IT infrastructure capability vs IT proactive stance [9], software modularity vs IT business partnership [12], and IS integration vs analytical ability [10]. Summarizing prior IT capability research, drawing on the wider capability-based research (e.g., [25, 26]), we apply a widely conceptualized notion of IT capability. We identify those two complementary views on IT capability as (1) reactive IT capability and (2) proactive IT capability.

*Reactive IT capability* supports and enhances work processes; for example, by managing and utilizing the IT infrastructure in order to support and enable the business [27]. Through flexibility of IT infrastructure, business operations become more efficient in their performance and more effective in implementing new solutions when facing uncertainty [28]. Therefore, utilizing proven and existing IT resources, as well as finding new ways for IT resources to best support current and future business operations, are essential for reactive IT capability [24]. Hence, we define reactive IT capability as an organization’s ability to acquire, deploy, combine, and reconfigure IT resources with a focus on supporting and enhancing work processes.

*Proactive IT capability* supports and enhances business strategy; for example, by implementing competitive measures driven by IT [29, 30]. Organizations search for new IT innovations and benefit from existing IT resources when following through on business opportunities [9]. Organizations use business analytics in order to monitor and analyze market data as part of their proactive IT capability [31]. We therefore define proactive IT capability as an organization’s ability to acquire, deploy, combine, and reconfigure IT resources with a focus on supporting and enhancing business strategies.

Our conceptualization of IT capability is aligned toward a value-oriented view that relates IT capabilities to their value outcomes. IT can either be characterized as a supporter or driver of business value [32]. Our description of IT capability is agnostic to previous typologies based on IT’s structure and form (e.g., classification into IT infrastructure, IT management, IT personnel). Since contemporary IT phenomena transcend organizational functions and are not restricted to traditional IT departments, we argue our view is timely and resonates well with the fusion of IT and business [33–35].

To provide conceptual clarity and avoid confusion, we explicitly distinguish reactive and proactive IT capability from another prominent dichotomous view of IT capability, namely, IT ambidexterity. IT ambidexterity represents balancing the two seemingly conflicting goals of exploiting existing IT solutions (IT exploitation) and exploring new IT solutions (IT exploration) [24]. We suggest there are three important differences. First, the ambidextrous view focuses on specific aspects that cover an organization’s ability to cope with change in situations of tension that result from scarce IT resources [36]. In that sense, IT ambidexterity focuses on innovation outcomes, for example, the development of incremental improvement and radical innovation during new product development [37]. Our conceptualization of IT capability seeks to cover a broader range of activities inside the organization and is not limited to specific situations of coping with change and achieving innovation outcomes. Second, IT ambidexterity provides only a partial representation of IT capability, which includes acquisition, deployment, combination, and reconfiguration of IT resources, whereas IT ambidexterity focuses on the acquisition and experimentation of new and existing resources. Third, reactive and proactive IT capabilities shape how the IT function is positioned relative to the business. They describe how the IT function generates value for business. Thus, they are formulated in relational terms to their desired outcomes. In contrast, IT ambidexterity is presented as a composite measure that results from a combination of successful exploitation and exploration of IT resources.

## Explanations for the effects of IT capability on organizational agility

Organizational agility is the ability to swiftly change businesses and business processes beyond the normal level of flexibility to effectively manage unpredictable external and internal changes [38]. While both reactive and proactive IT capability have been found to improve organizational agility (e.g., [5, 24, 39]), the reasons for the observed effect differ in respect of reactive IT capability and proactive IT capability. The literature presents us with three central reasons for the effect of IT capability on organizational agility that we refer to as (1) identifying, (2) processing, and (3) transforming.

*First*, IT capability helps organizations to identify responses to and opportunities arising from its changing environment and to learn from experiences as the organization evolves. Reactive IT capability assists the organization’s knowledge management, which assists in identifying the best responses to changing situations. In addition, reactive IT capability facilitates the creation of fluid structures that make it easier for organizations to adapt in changing environments. For example, IT planning skills align planning processes, develop reliable and cost-effective applications, and support business needs, resulting in sharing and assimilation of knowledge, resource reconfiguration, and the identification of business and resource needs that improve its operational agility [40]. Proactive IT capability allows an organization to identify and assess new opportunities and their value. Opportunities might be, for example, an emerging technology that impacts the business model or service offerings. Moreover, organizations can benefit from their IT to facilitate learning about their competitive landscape. For example, IT-enabled sensing capability facilitates monitoring of competitors and ensures customer feedback is received and analyzed in order to improve management decisions [41].

*Second*, IT capability improves organization’s information processing. This helps organizations to sense and respond to changes more effectively, thus improving their organizational agility [42]. With a focus on the operational level, reactive IT capability has been suggested to increase information flows within and across organizational units. Thus, it allows organizations to respond to changes more effectively. For example, IS integration increases the information flow within organizations and across distribution channels, allowing organizations to respond quickly to market opportunities [10]. Proactive IT capability provides seamless and consistent access to data, which allows organizations to have a better sense of their environment. Furthermore, proactive IT capability increases the transparency within organizations for continuous product innovation. For example, IT applications provide seamless and consistent access to organizations’ customer, production, order, and market data. Organizations also benefit from this data by being able to quickly sense and analyze their customers’ existing and latent needs [43].

*Third*, clever use of organizations’ IT resources helps organizations to transform their business processes in order to improve their agility [44]. Reactive IT capability helps organizations to use and benefit from their IT resources to meet changing business needs. For example, a globally integrated infrastructure provides a platform to generate digital options and assist the organization in accessing, synthesizing, and exploiting knowledge, and to cope with unexpected changes, respond to disruptions in supply and demand, and rapidly implement new IT-enabled offerings or initiatives [9]. In a similar vein, organizations might use their IT resources to translate innovative responses into business processes. For example, a proactive IT stance enables an organization to rapidly identify and select opportunities with IT innovations to address changing information needs that are in line with a changing business strategy [9]. Table 1 presents an overview of the key explanations for the relationship between reactive and proactive IT capability and organizational agility (see S1 Table for list of study examples).

**Table 1:** Explanations for the relationship between IT capability and organizational agility.

IT Capability	Identifying	Processing	Transforming
**Reactive IT Capability**	Improve knowledge management in order to identify the best response to a changing situation and facilitate the creation of a fluid structure for organizational adaptation in changing environments (e.g., [40]).	Increase information flows within and across units in order to increase responsiveness (e.g., [10]).	Use and benefit from IT resources to meet changing business needs by adjusting business processes (e.g., [45]).
**Proactive IT Capability**	Identify and assess new opportunities (e.g., emerging technology that impacts the business model or service offerings) and their value (e.g., [9]).	Provide seamless and consistent access to data, thus improving environmental sensing and transparency for continuous product innovation (e.g., [46]).	Use IT to translate innovative responses into business processes (e.g., [10]).

## Explanations for the effects of IT capability on organizational performance

IT capability also has direct effects on organizational performance [11, 19, 47]. The two central justifications for the relationship between IT capability and performance in literature reflect a supporting and a driving role. Reactive IT capability has a supporting role for business, that is, it supports business functions, which in turn, increases performance. For example, timely information availability, processing, and utilization support business functions in their work processes [29]. Furthermore, as reactive IT capability increases, organizational outcomes also increase, which suggests that IT is a source of business value and positive performance implications. For example, if organizational processes can be adapted with swiftness, robustness, and flexibility, organizations can achieve higher productivity [22].

By contrast, proactive IT capability follows a different logic and has a driving role for business. Proactive IT capability has been suggested to drive innovation through learning, alignment, partnerships, and trust [48]. Proactive IT capability enables the organization to rapidly sense and respond to changes and arrive at new IT-enabled value propositions (e.g., [41, 49]). IT is seen as a driver, rather than a supporter, of business.

Table 2 presents an overview of both explanations for the relationship between reactive and proactive IT capability and organizational performance (See S2 Table for list of study examples).

**Table 2:** Explanations for the relationship between IT capability and organizational performance.

IT Capability	Explanation
**Reactive IT Capability**	Increased reactive IT capability supports the business function through • Timely information availability, processing, and utilization (e.g., [29]); • Improved swiftness, robustness, and flexibility of digitized business processes (e.g., [22]); and • Strategic flexibility to refocus on resources (e.g., [43]).
**Proactive IT Capability**	Increased proactive IT capability drives business value through • IT business partnerships that increase trust between IT groups and business units by sharing risk and responsibility of IT applications (e.g., [48]); • Rapidly sense and respond to market changes and shifts in customer and supplier needs, improve capitalizing on the market by means of new or improved value propositions through focused deployment (e.g., [41, 49]).

## Research method

We conducted a confirmatory meta-analysis on reported effects sizes [16]. We tested and compared the effects of reactive and proactive IT capabilities, using random-effects models [50]. We opted for a meta-analytical research approach for multiple reasons. First, a meta-analysis allows us to systematically synthesize and summarize previous empirical studies [51, 52]. Second, we can identify more reliable correlations and identify more robust effect sizes between the variables (compared to collecting primary data through an empirical study). Third, we account for inadequacies introduced by individual studies (e.g., sampling bias), while simultaneously increasing the statistical power by relying on the empirical data from multiple studies [16, 50]. In line with prior studies (e.g., [53]), we start by describing our sample, which is followed by the coding and measurements and the analytical approach thereafter.

### Sample

**Fig 1:**
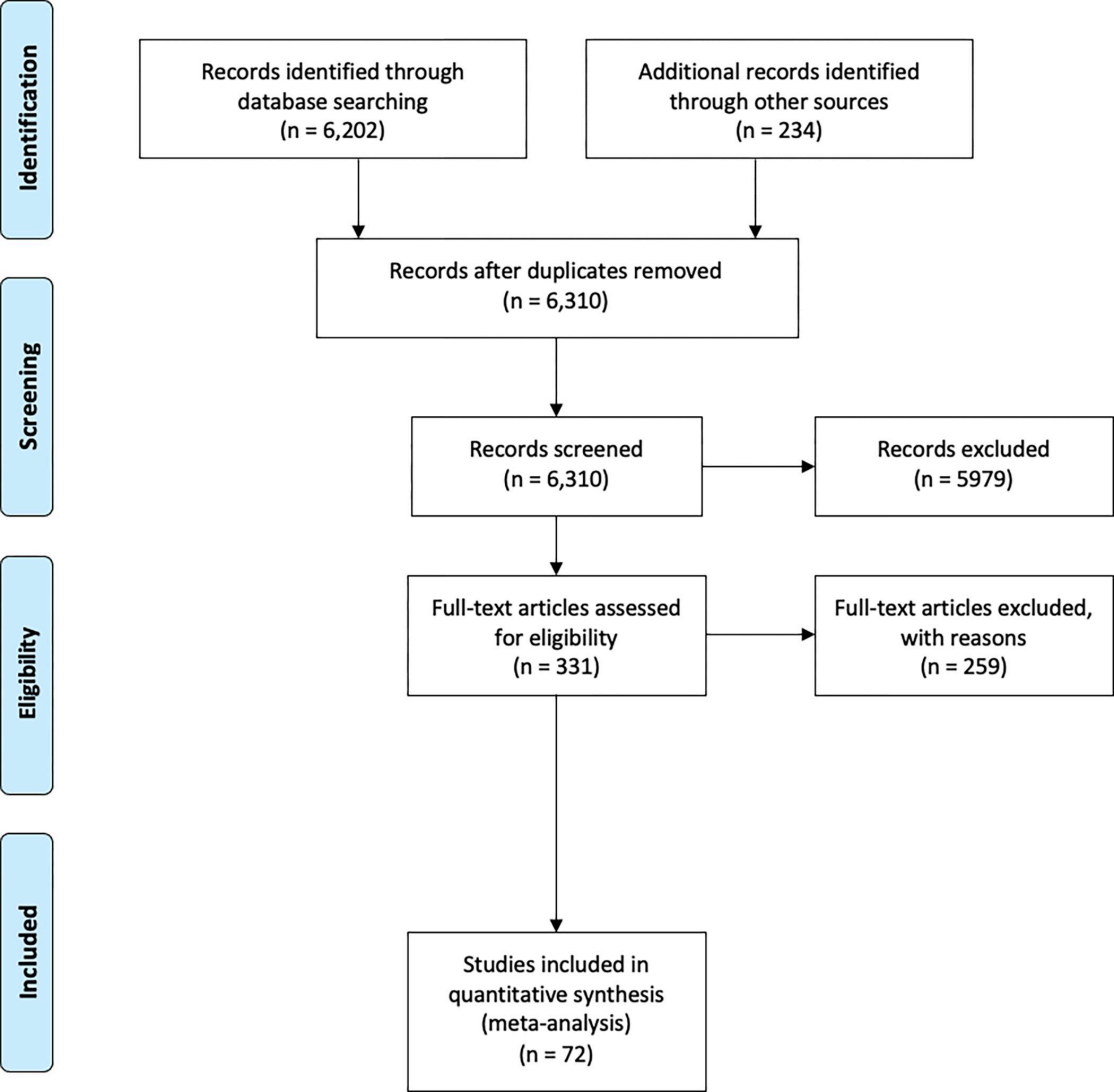
Overview of systematic review process.

We used multiple search strategies to identify relevant articles for organizational agility and organizational performance within the broader management literature. An overview of our systematic review process is depicted in Fig 1.

*First*, we started with 9 seminal papers that we were initially aware of (i.e., [10, 12, 22, 29, 43, 47, 54–56]). *Second*, we conducted a comprehensive keyword-based search in several databases such as EBSCO, ProQuest ABI Inform, and Scopus, where we identified 6,202 hits (see S1 File for details). *Third*, we expanded the results (additional records identified in Fig 1), complementing them with articles identified in other studies (e.g., we used recent literature reviews such as [39]). Furthermore, we used the ProQuest dissertations and theses database to identify additional empirical evidence. In contrast to peer-reviewed journals and conference proceedings, dissertations and theses have a higher tendency to include and report negative results–thus allowing us to mitigate a possible publication bias in our results. *Fourth*, we removed duplicates, screened the remaining results and excluded studies that did not meet minimal criteria (e.g., completed research, thematic relevance, published in English; see S1 File for a detailed list of criteria).

*Fifth*, we assessed the full text of studies for eligibility by using the criteria described below. Using this preliminary dataset, we conducted forward and backward searches in May 2021 in order to identify additional studies.

We included empirical studies that investigated IT capability using quantitative research methods (e.g., surveys, secondary data analysis, or experiments). Specifically, we determined the following minimum requirements for the relevance of each article (eligibility criteria). The study needed to report (1) a correlation (or other values that can be transformed into correlations; cf. [57]) between IT capability (or subconstructs thereof) and organizational agility or performance, (2) the measurement items for the constructs of relevant correlations, and (3) the sample size. If a quantitative study did not report on required information, we contacted the authors of the original study. If insufficient information for a study was provided, we excluded the study from our sample.

Furthermore, we accounted for studies based on overlapping datasets. A dataset was considered overlapping when the sample size was the same, sample descriptions where similar, overlapping reported correlations were identical, and the same measurement instrument was used. In these cases, we documented each relevant correlation only once, preferably from the study that contained or reported more information. Moreover, we distinguished studies with and without key information bias by capturing the number of responses and their respondents. All data were stored in a research database using MySQL. The final sample included a total of 72 quantitative empirical articles out of which 62 contained measures of reactive or proactive IT capability (i.e., the IT capability measurements of 10 relevant articles could not be categorized as either reactive or proactive). S3 Table presents an overview of these articles.

### Coding and measurement

We identified two possible issues related to the correct measurement of variables, referred to as the jingle-jangle fallacy [58]. First, studies may use the same terminology when measuring different phenomena (jingle). For example, studies may not operationalize IT capability as an organizational behavioral variable, but rather as a characteristic of the infrastructure. We excluded these studies from our analysis as the operationalizations were incommensurable with our definitions (e.g., when measuring inventory agility, such as in study [59]). Second, studies may use different terminology when measuring the same phenomenon (jangle). For example, studies related to IT competencies closely resemble the notion of IT capability (e.g., [3]). We included these studies as part of our eligibility assessment.

Our key constructs formed a substantial part of our concept matrix, guiding our coding process. We used the initial set of seminal articles to test and improve the inter-rater agreement during the coding process. Thereafter, the set of papers was split into two parts each of which was processed by its own dedicated coder. The coding was done in batches of 10–20 articles, after which alignment and workshop sessions were conducted with both coders to discuss ambiguous and exceptional cases. Furthermore, challenges encountered during the coding process were discussed and aligned between the coders.

The following section describes each construct and its measurements (see Table 3 for an overview). We conceptualized IT capability as being constituted by reactive IT capability and proactive IT capability. We measured reactive IT capability by means of scales that address IT infrastructure capability (e.g., [9]), IT development capability (e.g., [55]), and capabilities relating to operational IT-business alignment (e.g., [60]). We measured proactive IT capability by means of scales that address IT strategy (e.g., [61]), business analytics (e.g., [10]), business partnerships (e.g., [61]), and strategic capabilities related to IT-business alignment (e.g., [54]).

**Table 3:** Measurements of the focal constructs included in the meta-analysis.

Constructs and Measurements	Study Examples
**Reactive IT Capability**	
*Measured by scales addressing capabilities related to IT infrastructure*, *IT development*, *and operational IT-business alignment*.	[9, 55]
**Proactive IT Capability**	
*Measured by scales addressing capabilities related to IT strategy*, *business analytics*, *business partnership*, *and strategic IT-business alignment*.	[10, 61]
**Organizational Agility**	
*Measured by scales addressing an organization’s ability to adjust its operations in a reactive manner and/or to strategically embark on opportunities related to markets and customers*.	[9, 10, 29]
**Organizational Performance**	
*Measured by scales related to internal performance indicators (e*.*g*., *cost*, *cycle time*, *and efficiency of operations*.*) or an organization’s financial*, *product market*, *and shareholder return performance (e*.*g*., *return on investment*, *revenue*, *profitability*, *sales*, *profit*, *growth*, *and general success)*.	[22, 24, 29, 47, 62]

Organizational agility consists of sensing and responding capabilities. We measured agility by means of scales that address an organization’s ability to adjust its operations; for example, scales relating to adaptive agility (e.g., [29]), adjustment agility [9], and responding capability (e.g., [10]). We also made use of scales that address an organization’s ability to strategically embark on opportunities related to markets and customers. Accordingly, we incorporated measures for market capitalizing agility (e.g., [9]), entrepreneurial agility (e.g., [29]), and sensing capability (e.g., [10]).

Organizational performance was measured on the basis of indicators such as cost, cycle time, and efficiency of operations (e.g., [24]), as well as scales that address an organization’s financial, product market, and shareholder return performance (e.g., return on investment, revenue, profitability, sales, profit, growth, and general success; e.g., [47])

We extracted the following data from each article included in our final dataset. We documented bibliographic data (e.g., author, year, title, and outlet), and methodological data (e.g., research method, sample size, industry, respondents, date of data collection, and recruitment method). For each study, we extracted the correlations between the variables within our model, reliability scores, measurement items, and the items method type [63].

### Analytical approach

Our analysis consisted of two steps. First, we used meta-analytical guidelines to estimate the effect sizes of individual relationships using random effects models [50]. We made our calculations using the metaSEM package in R [64]. In the light of the existing criticism of confidence intervals in meta-analysis, we reported 80% credibility intervals [50]. First, we calculated the four meta-correlations for our main constructs–that is, reactive IT capability in relation to agility and performance, as well as proactive IT capability in relation to agility and performance.

Second, we used two additionally coded variables (i.e., industry and single vs multi-source studies) to conduct robustness checks of our findings. Since studies with extreme sample sizes, both large and small, can distort results, we checked all studies for exceptional cases. The sample sizes of our selected studies vary from 63 to 686, suggesting no extreme cases.

## Results

### Meta-analytic correlations

We estimated the central correlations between reactive IT capability, proactive IT capability, organizational agility, and organizational performance. Table 4 presents the results of the meta-analyses. We reported the number of datasets included in each correlation (k), the estimated correlation (r_+_), the standard error (S.E. _r+_), and the 80% credibility interval (CV_r+_ low/high). The results suggest medium correlation sizes. However, there was no statistically significant difference between the effect of reactive IT capability (r_+_ = 0.39, k = 34, 95% Confidence Interval (CI) [0.34, 0.44]) and proactive IT capability (r_+_ = 0.38, k = 21, 95% CI [0.31, 0.45]) on organizational agility (z = 0.68, p = 0.25). Moreover, there was no statistically significant difference between the effect of reactive IT capability (r_+_ = 0.31, k = 43, 95% CI [0.26, 0.37]) and proactive IT capability (r_+_ = 0.33, k = 25, 95% CI [0.27, 0.40]) on organizational performance (z = 1.11, p = 0.13). Since these results aggregate multiple studies from different authors, industries, and countries, they are an adequate representation of empirical findings. We reported Tau^2^ and the I^2^ (Q-statistics). Tau^2^ indicates the between-study variance, while I^2^ indicates whether the variance across studies is due to heterogeneity. As the I^2^ scores indicated a substantial to considerable level of heterogeneity, we investigated further for potential moderating effects.

**Table 4:** Overview of meta-correlations.

Correlation	k	n	r_+_	S.E._r+_	CV_r+_ low	CV_r+_ high	Tau^2^	I^2^ (Q statistic)
**Organizational Agility**								
**ITCR–OA**	34	9401	0.39	0.03	0.27	0.51	0.02	0.86
**ITCP–OA**	21	5267	0.38	0.04	0.25	0.51	0.02	0.86
**Organizational Performance**								
**ITCR–OP**	43	7078	0.31	0.03	0.17	0.45	0.03	0.89
**ITCP–OP**	25	3870	0.33	0.03	0.20	0.46	0.02	0.86

*Note*. k = number of independent datasets; n = observed total sample size; r_+_ = random-effects average correlation; S.E. _r+_ = standard error of r_+_; CV_r+_ = credibility interval of r+; ITCR = reactive IT capability; ITCP = proactive IT capability; OA = organizational agility; OP = organizational performance.

### Robustness checks

Given the suggested heterogeneity (see I^2^), we investigated the following moderators to check the robustness of our results (see Table 5): industry as an indicator for environmental changes, and single vs multi-source studies as an indicator for study quality. For the industry moderator, we categorized each data sample as either manufacturing industry, service industry, or cross-sectional industry. The services industry is not reported in our results due to the limited number of studies for each reported correlation. For study quality, we distinguished between studies that relied on a single data source from those that relied on multiple data sources, often accounting for potential biases.

**Table 5:** Overview of meta-correlations by moderator.

Moderator	Correlation	k	n	r_+_	S.E._r+_	CV_r+_ low	CV_r+_ high	Tau^2^	I^2^ (Q statistic)
**Single-Source**	ITCR-OA	28	5927	0.40	0.03	0.28	0.52	0.02	0.87
	ITCP-OA	15	2525	0.39	0.05	0.24	0.54	0.03	0.87
	ITCR-OP	33	7249	0.33	0.03	0.21	0.45	0.02	0.85
	ITCP-OP	19	3750	0.34	0.04	0.22	0.46	0.02	0.87
**Multi-Source**	ITCR-OA	6	1151	0.34	0.05	0.26	0.42	0.01	0.74
	ITCP-OA	6	1345	0.36	0.06	0.24	0.48	0.02	0.83
	ITCR-OP	10	2152	0.25	0.08	0.04	0.46	0.06	0.92
	ITCP-OP	6	1517	0.31	0.07	0.19	0.43	0.02	0.85
**Manu-facturing**	ITCR-OA	8	1712	0.30	0.08	0.12	0.48	0.05	0.92
	ITCP-OA	5	1033	0.25	0.10	0.08	0.42	0.04	0.90
	ITCR-OP	13	2718	0.27	0.03	0.19	0.35	0.01	0.71
	ITCP-OP	6	935	0.22	0.06	0.11	0.33	0.02	0.74
**Cross-sectional**	ITCR-OA	24	5087	0.43	0.02	0.35	0.51	0.01	0.75
	ITCP-OA	15	2667	0.43	0.03	0.35	0.51	0.01	0.78
	ITCR-OP	27	6341	0.34	0.04	0.17	0.51	0.04	0.92
	ITCP-OP	17	4081	0.37	0.04	0.25	0.49	0.02	0.88

Note. k = number of independent datasets; n = observed total sample size; r_+_ = random-effects average correlation; S.E. _r+_ = standard error of r_+_; CV_r+_ = credibility interval of r_+_; ITCR = reactive IT capability; ITCP = proactive IT capability; OA = organizational agility; OP = organizational performance.

Furthermore, we tested for publication bias by comparing correlations published in scientific outlets (such as conferences and journals) with those published in dissertations. Due to the limited number of dissertations, we collapsed reactive and proactive IT capability into a single construct, testing two relationships: IT capability toward agility and IT capability toward performance. For the effect of IT capability on organizational agility, there was no statistically significant difference (z = 0.42, p = 0.34) in scientific outlets (r_+_ = 0.40, k = 44, 95% CI [0.36, 0.45]) and dissertations (r_+_ = 0.38, k = 2, 95% CI [0.30, 0.47]). For the effect of IT capability on organizational performance, there was a statistically significant difference (z = 2.04, p = 0.02) in scientific outlets (r_+_ = 0.34, k = 54, 95% CI [0.29, 0.39]) and dissertations (r_+_ = 0.26, k = 4, 95% CI [0.17, 0.34]). We therefore conclude that publication bias is not an issue in our data toward agility but shows a small effect in our data toward performance.

The results in Table 5 suggest that our initial findings are robust. The effect sizes of reactive and proactive IT capability in relation to organizational agility and performance are robust across multiple groups. There were only few exceptions. For example, the relationship between reactive IT capability (r_+_ = 0.25, k = 10, 95% CI [0.10, 0.41]) and proactive IT capability (r_+_ = 0.31, k = 6, 95% CI [0.17, 0.44]) toward performance for studies with multi-source shows the biggest difference (z = 1.94, p = 0.03).

## Discussion

In this study, we set out to investigate how reactive and proactive IT capability differ in their effects on agility and performance. Although prior research suggests the contrary, our results based on a meta-analysis of empirical studies indicate that there is no statistically significant difference in the effects of reactive and proactive IT capability on agility and performance.

Our results are surprising, given the widespread notion of two different IT capabilities in the literature, namely reactive and proactive IT capability. For example, prior studies distinguished between IT support for functionality-related competency and IT support for market-access competency [43], IT infrastructure capability and IT competencies [29], and internally-focused and externally-focused IT capabilities [49]. While prior studies often limited their investigation to either one IT capability concept (e.g., [65]), or the interaction effect of both IT capabilities [24, 43], we investigate reactive and proactive IT capability as distinct concepts. However, our analysis of empirical studies shows that we cannot distinguish the effects of IT capability toward agility and performance based on reactive and proactive IT capability alone, suggesting an IT capability fallacy.

In the following, we discuss these results and explore four possible explanations for their reconcilability with results of individual studies. First, reactive and proactive IT capability might be attributed to different mechanisms in line with prior research. For the relationship between IT capability and performance, researchers contemplate on business supporting and business driving mechanisms (e.g., [34]). For the relationship between IT capability and agility, researchers refer to reactive IT capability as a platform to generate digital options to implement new IT-enabled offerings or initiatives while proactive IT capability as an enabler of business-IT synergies that lead to the translation of innovative responses and radical change to processes and IS [9]. Notwithstanding the assumption of different mechanisms, effect sizes of reactive and proactive IT capability might still be similar. However, we suggest that this explanation is unlikely, as different mechanisms are likely to lead to different results when compared across groups. For example, proactive IT capability to support and enhance business strategy could be more important in volatile and turbulent environments [29] affecting both, agility and performance. Since volatility and turbulence has been attributed to different industries [66], we would expect different results when accounting for industry as a moderator. Since our results do not support an explanation through different mechanisms, we rule out this explanation.

Second, another possible explanation for our results is an unobserved mediation from reactive and proactive IT capability toward agility or performance. Varying direct and indirect effects of (reactive and proactive) IT capability could sum up to the same overall effect sizes. When these effects are investigated more closely, we could observe a stronger mediating effect for one IT capability than the other. If the direct effects have the opposite pattern, the overall effect size of reactive and proactive IT capability could be the same. For example, prior studies suggest that organizational agility mediates the relationship between (reactive and proactive) IT capability and organizational performance. Assuming this mediation, reactive and proactive IT capability have both a direct effect toward performance and an indirect effect that is mediated through agility. Given the importance of an organization’s ability to effectively combine IT resources for performance through business support [18] and IT-enabled ability to sense in agility research [10, 31], we now consider different direct and mediating effects. For example, reactive IT capability has a stronger direct effect on performance (e.g., β = 0.3 for the direct effect) with a weaker mediating effect of agility in this relationship (e.g., β = 0.1 for the indirect effect) in contrast to a weaker direct effect of proactive IT capability (e.g., β = 0.1 for the direct effect) and a stronger mediating effect of agility in such relationship (e.g., β = 0.3 for the indirect effect). In this example, the overall effect size would be the same, but we would also expect to see these differences reflected in the relationship between IT capability and agility. Since our results do not support this explanation, we also rule out this explanation.

Third, there might be temporal differences in the effects of reactive and proactive IT capability that are not visible in our classical variance-based analysis. The investigation of temporal effects has only recently received more attention in IS (e.g., [67, 68]) and research on temporal effects of IT capability remains scarce. Exception exists with econometric approaches that employ archival data (see for example, [49] from our sample). The interest in time-dependent research resonates well with an upsurge in related fields such as management research [69]. Recent advances in the field of dynamic capability suggest that different capabilities have different temporal effects [70]. Building on this notion, we suggest that the benefit of reactive and proactive IT capability for organizations has different temporal effects in latency and sequence. For example, we suggest that for change requirements, organizations benefit immediately through reactive IT capability, which refers to the ability to react to change through IT on a business process level (which in turn has a direct positive effect on performance indicators). By contrast, proactive IT capability affects the strategic level that first needs to be broken down and translated to the operational level before it can impact performance. While our data does not allow us to conduct a time-dependent analysis, our results provide preliminary evidence for this proposition through generally higher effects of (reactive and proactive) IT capability on agility than on performance.

As a fourth explanation, the effects of reactive and proactive IT capability might be more complex than previously assumed. The different effects of organizational capabilities manifest only in combination with several other moderators as suggested by research from IS and management [71, 72]. For example, complex interactions may exist between IT (e.g., reactive and proactive IT capability), dynamic capabilities (e.g., agility), and environmental (e.g., turbulence) and organizational conditions (e.g., organization size and age). While our moderator analysis supports the robustness of our results, we suggest that more complex interactions might be at work. Three-way or four-way interactions are difficult to grasp with classical reductionist modeling approaches [73]. Research on IT-enabled capabilities that enable organizations to cope with change in dynamic environments suggests these multi-way interactions [71], which can be investigated using configurations. Configuration theory assumes that different configurations exist that can equally enable desired outcomes (equifinality), such as agility and performance. For example, a recent study identified successful organizational configurations that enable organizational agility through communication technology and business intelligence [31]. Hence, the set-theoretic approach [74] is an appropriate inquiry instrument to investigate complex interactions and equifinality in the context of IT capabilities, organizational agility, and performance. Set-theoretic approaches have been recently suggested for IS research to investigate complex digital phenomena [75]. Next, we use temporal differences and complex interactions as a point of departure to identify new directions for future research.

## Future research directions

**Fig 2:**
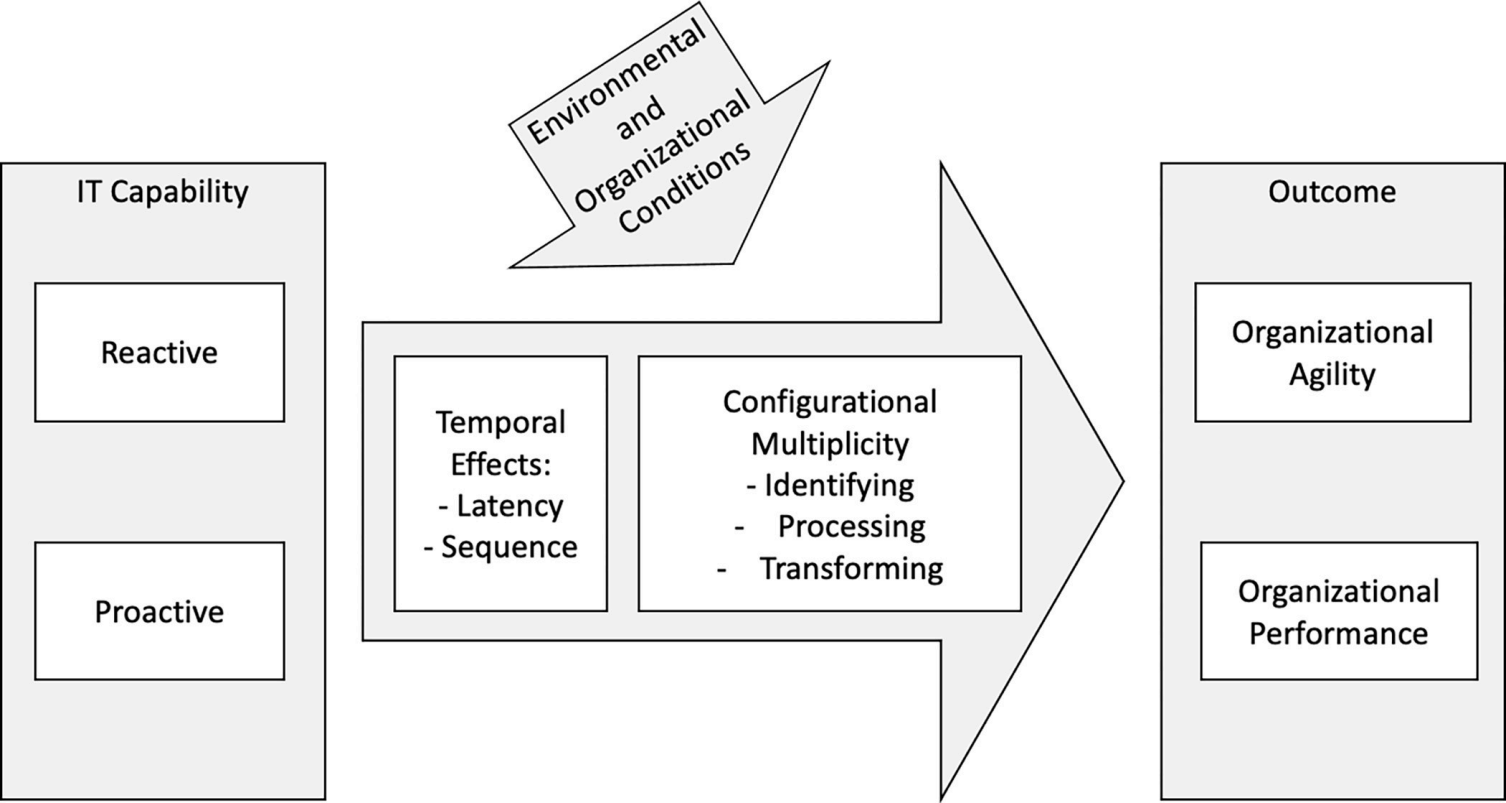
The nomological network of relationships between IT capability and organizational agility and performance.

Our findings have important implications for future capability-based research on IT and agility (and their organizational outcomes), which can benefit from adopting a temporal and configurational view. Fig 2 depicts the relationship between IT capability and organizational outcomes such as agility and performance in a nomological network. Complex interactions, which we elaborate on in the following, are based on temporal effects and a multiplicity of configurations embedded in environmental and organizational conditions such as environmental turbulence and organizational size and age. We identify four important areas that future IS research on IT capability can address: i) the latency of temporal IT effects, ii) the sequence of temporal IT dependencies, iii) IT configurations related to identifying, processing, and transforming, and iv) configurations related to IT as a supporter and driver of business. Table 6 presents an overview of these directions for future research.

**Table 6:** Overview of future research directions.

View	Important Areas for IS Research to Develop	Potential Research Questions
**Temporal**	Latency: Examine temporal effects of reactive vs proactive IT capability.	• How do lagged values of reactive and proactive IT capability influence agility and performance? • How does the rate of changes in IT capability affect changes in agility and performance? • How does the relationship between reactive and proactive IT capability and agility and performance vary over time? • What is the effect of the function of reactive and proactive IT capability on trends in agility and performance?
	Sequence: Identify temporal dependencies between different types of IT capability.	• What is the sequence of the subdimensions of IT capability, agility, and performance? • How does the process of enacting IT capabilities unfold concerning organizational agility and performance?
**Configurational**	Configurational Multiplicity: Examine how effects related to identifying, processing, and transforming interact.	• Which successful configurations exist to achieve beneficial organizational outcomes through IT capability? • What is the explanatory overlap (raw and unique coverage) of such configurations? • What contextual factors complement or compete with prior configurations for achieving agility?
	Theoretical Multiplicity: Identify causal recipes for IT capability as a multidimensional construct.	• What are the causal recipes for the relationship between IT capability and organizational agility and performance?

### Latency of reactive and proactive IT capability

Our first area for future research focuses on the latency of temporal effects; in other words, the fact that some effects take longer to generate a certain outcome than others [76]. We draw on recent results from dynamic capability [70] that offers a point of departure for the latency of IT capability’s temporal effect. Girod and Whittington [70] compared two effects of organizational reconfiguring and organizational restructuring. While restructuring requires changing fundamental principles of organizational design, it has delayed positive effects. Reconfiguring–also sometimes referred to as “patching”–refers to unit changes within existing organizational principles and has delayed negative implications. Similarly, we suggest that reactive and proactive IT capability have differing temporal effects that an organization needs to balance for sustainable performance gains. Hence, we suggest that the effect of reactive IT capability is more immediate and can quickly generate an effect to enhance organizational agility and performance outcome, such as process efficiency. Proactive IT capability, on the other hand, is less immediate and requires more time to influence strategic indicators. Changes in IT strategy, for example, have longer implementation cycles than changes to work processes. Acknowledging this temporal latency and investigating it could be of significant interest to IS researchers, who could pose the following question: *How do lagged values of reactive and proactive IT capability influence agility and performance*?

IT capability is also subject to changes [77]. For example, a new hiring strategy can influence how members use and benefit from IT. While an existing workforce may struggle to benefit from the latest trends in artificial intelligence (AI) or data analytics, finding prospective managers that have such expertise to fill central roles can have a lighthouse effect, in that the new manager can showcase the importance of such skills and how they benefit the organization’s ability to use IT. As such, the new manager brings new ideas relating to current business practices, and new IT capability can lead to innovation and performance gains. Alternatively, a corporate training program could equip the existing workforce with these needed skills to enhance the organization’s IT capability. While both scenarios increase the IT capability of an organization, one takes more time to implement than the other. For example, hiring new managers can be done relatively quickly, whereas the development and rollout of a training program takes more time. While the effect of new hires might be more immediate, their overall effect toward organization outcomes might be limited in contrast to training programs, which may require more time to implement but often scale better to larger organizations. IT capabilities are expected to affect organizational outcomes, yet little is known about how the rate of changes in IT capability impacts such organizational outcomes. This is particularly interesting when considering that hiring new managers and launching a training program incur costs that also negatively impact performance. However, it is still unclear how the rate of changes in IT capability influences organizational outcomes. Consequently, IS researchers might ask: *How does the rate of changes in IT capability affect changes in agility and performance*?

We also know that IT strategy changes over time [78]. A changing IT strategy provides new directions and priorities that impact proactive IT capability. For example, an IT strategy that aligns well with the business strategy, addresses competitive pressures, technological advances, and new market opportunities is more successful. By contrast, a poorly formulated strategic IT vision results in many strategic actions remaining ambiguous, leading the organization to rely more on its reactive IT capability. Hence, changing priorities within an organization affects the relationship between proactive and reactive IT capability and agility and performance. With a changing effect over time, IS researchers might ask the following: *How does the relationship between reactive and proactive IT capability and agility and performance vary over time*?

Going one step further, the strength of the relationship between IT capability and organizational outcome may also impact future changes in organizational outcome. For example, organizations may suffer from a cold-start problem when it comes to creating and evolving their IT capability based on recent technological advances. The cold-start problem [79] refers to the inability of an organization to benefit from IT capability, when a critical level of needed resources has not yet been attained. For example, a corporation may hire an AI engineer to explore the opportunities of AI for its business. While the new engineer has good intentions and makes consistent progress, the development of new infrastructure and organizational IT capability will be rather slow. By contrast, when the same organization hires a team of AI engineers the progress will be quicker, and the organization is more likely to attract additional resources. Therefore, the degree to which an organization benefits from IT capability can relate to future changes in agility and performance. As a result, IS researchers might ask the following question: *What is the effect of the function of reactive and proactive IT capability on trends in agility and performance*?

Previous research on strategic management [70] and strategic change [69] suggest different temporal effects such as latency, rate of change, and time events, influence organizational performance. Simultaneously, researchers in management and organizational studies call for more research investigating temporal effects (e.g., [69, 80]). New methods for analyzing time-dependent data are needed [81]. While few examples exist (e.g., [68]), studies collecting time-dependent data on organization’s use of IT are still scarce.

### Sequence of reactive and proactive IT capability

Our second area for future research emphasizes temporal dependencies. Proactive and reactive IT capability may have an implicit sequence. For example, proactive IT capability supports and enhances business strategy. Nevertheless, changes at the strategic level need to be broken down to the operational level before they can have an impact. Consequently, actions resulting from proactive IT capability require reactive IT capability to be enacted. Such reactive IT capability will first improve operational performance measures, such as process efficiency, before impacting higher-level key performance indicators defined in the business strategy. This suggests that operational variables may mediate the effect at the strategic level. IS researchers might investigate these sequences further by asking: *What is the sequence of the subdimensions of IT capability*, *agility and performance*?

In addition, reactive IT capability influences and supports work processes. Multiple changes at this level may indicate a fundamental problem that cannot be solved by changes in the work processes. Rather, proactive IT capability is needed to trigger more fundamental changes to the organization’s strategy or principles to achieve more radical performance gains. These patterns can be investigated and identified using, for example, process mining and analytics [82]. Tracing event chains can identify multiple instances of these sequences with a view to uncovering unique and iterative processes. Using such data helps IS researchers to advance theorizing about processes. We know different archetypes of process theories (evolutionary, dialectic, lifecycle, and teleological) have been considered in the context of organizational research [83]. Consequently, future IS researchers could investigate different sequences, their patterns, and explanations by asking the following question: *How does the process of enacting IT capabilities unfold concerning organizational agility and performance*?

This research area draws on prior calls for action to increase data-driven research in IS [84]. For example, one previous study has demonstrated a novel method for using digital trace data while benefiting from contextual information [85]. Contextual details of process data assist researchers in process theorizing. While process data are often limited to events and timestamps, the example demonstrates that contextual information, such as the actor, system, and location, allows researchers to identify and explore possible explanations through narrative networks–a weighted graph consisting of events (nodes) and their sequential relationships (edges; [86]).

### Configurational multiplicity of IT capability

A third area for future research builds on the configurational view, concerning the effect of either reactive or proactive IT capability on organizational agility, researchers have used three types of explanations (i.e., identifying, processing, and transforming). For example, IT capability can help *identify* new opportunities from IT or best responses to a changing situation. IT capability can also benefit from information *processing* provided by IT systems to increase the information flow or provide seamless and consistent access to data. An organization can also use IT to *transform* business processes in order to meet changing needs and translate innovative responses into business processes. However, we would expect that the benefits of each of these three are dependent on a multiplicity of factors. This situation suggests to investigate their configurational multiplicity–i.e., “the existence of multiple configurations of relevant factors for a given theoretical perspective” [75]–whereas previous research tended to adopt a “‘the more the better’ linear model” (see [87], p. 3). A configurational view allows us to investigate possible multiple interactions. Consequently, researchers might ask the question: *Which successful configurations exist to achieve organizational agility through IT capability*?

Following up on this question, we can distinguish between different successful configurations by their raw and unique coverage [88]. Raw coverage refers to the membership of cases in a configuration that achieves a certain outcome. Unique coverage refers to the membership of cases in a single configuration that achieves a certain outcome. In the case of explanations of how IT capability enables agility, we can investigate configurations of all three explanations (i.e., identifying, processing, and transforming). Depending on contextual factors, an organization may benefit from IT capability to enhance its information flow–for example, when the organization has strong value chains or requires a free flow of information within organizational boundaries. Hence, future research should further address the question: *What is the explanatory overlap (raw and unique coverage) of IT identifying*, *processing*, *and transforming configurations for agility*?

Given the possible contextual factors that affect the relationship between IT and agility and performance, a configurational view can provide much clarity when it comes to the results of previous research. For example, the importance of reactive and proactive IT capability might differ in dynamic environments and stable environments. Organizational research on agility has occasionally devoted attention to contextual factors such as environmental dynamism and industries; however, not in a systematic manner and not while evaluating the trade-offs of reactive and proactive agility in relation to agility and performance. While the previous questions focused on configurational multiplicity of the various important concepts, researchers might also investigate contextual factors that influence the (un-)successful outcomes by asking: *What contextual factors complement or compete with prior configurations for achieving agility and performance*?

Although previous research is unanimous that an increase in each effect of IT capability is beneficial, an investment in either reactive or proactive IT capability is accompanied by negative effects as well. Similar to agility [89], IT capability incurs costs through investments in infrastructure and human resources [17, 76]. Previous research has demonstrated that not all IT investments are equally important in situations where a set of contextual factors are present and multiple interaction effects are at play. For example, research has found that business intelligence and communication technologies can have different levels of importance for achieving agility when considering varying degrees of contextual factors such as organization size and environmental dynamism [31]. We suggest that multiple configurations for IT capability exist that either enable or constrain agility, depending on interactions with contextual factors. We find examples of such factors in the literature–for example, environmental dynamism (e.g., [29]) and industry (e.g., [40]).

### Theoretical multiplicity of IT capability

Our fourth area for future IS research suggests the application of the configurational view for theoretical multiplicity, that is, “the applicability of multiple theoretical perspectives” [75]. Theoretical multiplicity allows researchers to investigate conflicting roles of IT to derive successful outcomes. For example, previous research on the IT-performance relationship explains the varying effects of reactive and proactive IT capability and differentiates between a business-supporting and business-driving role of IT. Reactive IT capability is considered to *support* business functions, whereas proactive IT capability is referred to as a *driver* of the business.

From a configurational point of view, we would expect successful configurations: i) where IT is seen to support the business functions, and ii) where IT is seen as a driver of business value. However, given the omnipresence of IT, we would not expect configurations in which neither perspective is present. The configurational view also allows researchers to investigate the competing or complementary nature of these two roles based on the presence and absence of certain indicators. The competing or complementary effects may very well be driven by contextual factors, such as the environmental dynamics or the environmental complexity an organization must navigate.

While configurational analysis as in the form of set-theoretic approaches is a means to identify and describe successful configurations (grounded in empirical data and containing manifold contextual factors), theory development can be achieved by leveraging causal recipes. Causal recipes are “formal statements explaining how the causally relevant elements combine into configurations in ways to produce a target outcome” (see [87], p. 9). Causal recipes link different theoretical perspectives to ecologies of configurations. For example, we may explain configurations that rely on a supportive view of IT capability with a resource-based view of the firm, whereas we may use IT as a driver by building on dynamic capability theory. Future research can examine theoretical multiplicity by asking: *What are the causal recipes for the relationship between IT capability and organizational agility and performance*?

IS researchers could investigate the multidimensionality of IT capability and identify successful and unsuccessful configurations of IT capability by using configurational theory. Identified configurations allow IS researchers to advance theoretical understanding through the identification of causal recipes [75]. The process of theorizing can generally take place in accordance with the notions of deduction and induction. We suggest that an inductive view might be more suitable for future research on the IT-agility relationship since there is limited understanding of the three identified explanations. Hence, researchers need to (1) understand the phenomenon of interest (e.g., through meta-theories), (2) empirically analyze multiple configurations, and (3) interpret results and build new causal recipes. For the IT-performance relationship, on the other hand, we suggest a deductive approach, as previous research provides us with possible predictions. Thus, researchers need to (1) hypothesize causal recipes, (2) empirically analyze multiple configurations, and (3) map configurations and validate hypothesized causal recipes.

### Limitations

As with any piece of research, we acknowledge our study has limitations. First, our categorization of reactive and proactive IT capability measures was achieved by an inter-subjective process that is to certain degree subject to interpretations of the coders. However, we did not introduce new categorizations but relied on two dominant arguments of IT capability from literature and independently coded all categorizations on item level. Second, although we checked our results for publication bias, we cannot rule out that the effect of IT capability toward performance is biased since there is a limited number of dissertations available covering this relationship. Third, we cannot directly test the formulated recommendations as future research directions with our data (e.g., we do not have time-dependent data). However, we provide preliminary evidence based on our analysis that supports our reasoning.

## Conclusion

Information systems researchers have contributed substantial knowledge to reactive and proactive IT capability and their effects on agility and performance. We reviewed and synthesized these remarkable contributions to conduct our own meta-analytical investigation. Much to our surprise, we found an IT capability fallacy, that is, the overall effects of reactive and proactive IT capability on either agility or performance are not distinguishable. These findings remained robust while testing for different moderators such as industry and data source. We argued for confounding effects of a deeper theoretical nature.

Consequently, we developed four important areas for future research on the effects of IT capability: i) researchers should examine temporal differences in the effects of reactive and proactive capability, ii) they should investigate temporal dependencies between IT capabilities, iii) they should examine how different explanations for the effect of IT capability on agility helps to predict successful outcomes, and iv) they could benefit from the configurational view in developing new causal recipes–for example, on the relationship between IT and performance. We believe that these are important future research directions that assist researchers of IT capability to further advance our understanding of how an organization’s ability to use IT improves its organizational agility and performance.

Our results have important theoretical and practical implications. While IT capability has been seen traditionally as a unified concept, researchers have relied on a dichotomy to explain their varying effects toward agility and performance. While we find that both types of IT capability are important drivers for organizational agility and performance, our results suggest that this differentiation alone is not sufficient. Rather, we need to understand different configurations of IT capability in conjunction with other environmental and organizational conditions and their changes over time in order to predict agility and performance.

In a similar vein, our results have important implications for IT managers. The dichotomy of IT capability requires managers to reflect on their composition of reactive and proactive IT capability within their organization. Managers need to understand their interaction with additional contingencies and how they may change their composition over time when accounting for further contingencies, such as environmental turbulences, for agility and performance gains.

## Supporting information

S1 Checklist PRISMA 2009 checklist. (DOC)

S1 Table Examples of studies on the relationship between IT capability and agility. (PDF)

S2 Table Examples of studies on the relationship between IT capability and performance. (PDF)

S3 Table List of included studies, sample sizes, and correlations. (PDF)

S1 File Details for the computerized search. (PDF)
