# Supplementary material for: A meta-analysis on the effects of IT capability toward agility and performance: New directions for information systems research
Source: PLoS One. 2022 Oct 27;17(10):e0268761. doi: 10.1371/journal.pone.0268761 (PMC9612477; doi:10.1371/journal.pone.0268761)
Supplement: S1 Table — (PDF) [file pone.0268761.s002.pdf]

A meta-analysis on the effects of IT capability toward agility and performance: New directions for information systems research

S1 - Examples of studies on the relationship between IT capability and agility

Table S1 gives an overview of different reasons found in the literature for the effect of reactive and proactive IT capability toward organizational agility. The table presents exemplary studies in support of our conceptualization of reactive and proactive IT capability and their varying effects toward agility.

Table S1 – Overview of exemplary studies, their concepts and reasoning for the effects of reactive and proactive IT capability toward agility

Author (Year)	ITC Concept	Reasoning for ITCR-OA relationship	Reasoning for ITCP-OA relationship
**Breu et al. 2002** [1]	**ITCR:** Use of ICT	IT infrastructure allows the organization to create fluid, flexible, and adaptive structures within dynamic environments.	
**Fink and Neumann 2007** [2]	**ITCR:** IT-dependent system agility, (IT) behavioral capability	IS capability to adapt to changing requirements and to adjust the utilization of information resources in accordance with new information needs is required for using IT to react quickly and effectively.	
**Lu and Ramamurthy 2011** [3]	**ITCR:** IT infrastructure capability<br>**ITCP:** IT business spanning capability, IT proactive stance	A globally integrated infrastructure provides a platform to generate digital options and assist the firm in accessing, synthesizing, and exploiting knowledge as well as cope with unexpected changes, respond to disruptions in supply and demand, and to rapidly implement new IT-enabled offerings or initiatives.	Business-IT synergies enable translation of innovative responses and radical change to processes and IS. IT business partnerships support improvised and informal decision making. Proactive IT stance enables the firm to rapidly identify and select opportunities with IT innovations to address changing information needs that are in line with changing business strategy.

**Tallon and Pinsonneault 2011** [4]	**ITCR:** Software modularity, network connectivity<br>**ITCP:** IT business partnerships	IT flexibility through scalability and adaptability parallel the notions of reach and richness within the digital options.	IT business partnerships help communicate business strategy and IT-led capabilities and can impede agility.
**Roberts and Grover 2012** [5]	**ITCR:** Internal IS integration, External IS integration<br>**ITCP:** Analytical ability	IS integration increases the information flow within the organization and across distribution channels, allowing organizations to respond quickly to market opportunities.	Use of analytical tools allows organizations to identify and assess the value of customer-based market opportunities.
**Chakravarty et al. 2013** [6]	**ITCR:** IT infrastructure capability<br>**ITCP:** IT capability	Infrastructure or technical IT skills enable firms to defend against a wide range of disruptive IT-enabled scenarios and be resilient; whereas communication and coordination tools improve transactional processes.	IT capability help foresee a wide range of IT-enabled scenarios and rapidly respond to opportunities. Their facilitation of managerial cognition and sense making in turn help firms to leverage their organizational agility.
**Liu et al. 2013** [7]	**ITCP:** IT assimilation		Assimilating advanced IT applications helps the firm develop an integrated information flow with channel partners, which enables the firm to achieve rich content as well as reliable and real-time information across the supply chain to immediately identify qualified products that are suitable for its requirements.
**Lee et al. 2015** [8]	**ITCR:** IT exploitation capability<br>**ITCP:** IT exploration capability	IT exploitation to fully utilize and leverage existing IT resources to reap benefits from diverse operational activities supported through timely and optimal IT support and infrastructure.	IT exploration directs attention toward the emerging technologies, methodologies, and skills.
**Lee et al. 2016** [9]	**ITCR:** IT infrastructure, IT planning<br>**ITCP:** IT-based knowledge management	IT infrastructure facilitates innovation and continuous improvement, implements and expedites business activities and operations through quicker access to and sharing of data across business processes. IT planning skills align planning processes, develop reliable and cost-effective applications, and support business needs, resulting in sharing and assimilation of knowledge, resource reconfiguration, and identifying business and resource needs that improve its operational agility.	IT-based knowledge management facilitates learning and understanding of its status in the competitive landscape.
**Chen et al. 2017** [10]	**ITCR:** IT support for functionality-related competency<br>**ITCP:** IT support for market-access competency	IT support for functionality-related competency improves redesigning production processes to enhance the ability to react to environmental changes.	IT applications provide seamless and consistent access to an organization's customer, production, order, and market data. They benefit from this data to rapidly sense and analyze the customers' existing and latent needs.
**Ghasemaghaei et al. 2017** [11]	**ITCR:** Data tools fit, tasks tools fit, people tools fit	High fit allows quick and effective analysis and enhances understanding as well as response of employees.	
**Mikalef and Pateli 2017** [12]	**ITCP:** IT enabled sensing capability		IT-enabled sensing capability facilitates monitoring of competitors and ensures customer feedback is received and analyzed to improve management decisions.

**Ravichandran 2018** [13]	**ITCR:** Digital platforms	Digital platforms enable IT-based business innovations at a lower cost than competition and to connect with a diverse set of external entities. Therefore, bridging weak ties to span structural holes in interorganizational networks and thereby increase awareness.	
**Queiroz et al. 2018** [14]	**ITCR:** IT application orchestration capability	IT resources meet business process needs; failing to renew IT resources will prevent organizations from adapting to market changes.	
**Tsou and Cheng 2018** [15]	**ITCR:** IT capability	IT capabilities improve processing of information, assisting the organization to sense and respond to changes.	
**Ashrafi et al. 2019** [16]	**ITCP:** Business analytics capabilities		Business analytics increase information quality needed for sensing market changes, ensuring appropriate organizational decision-making, being creative and testing new ideas in a virtual environment, and increasing entrepreneurial decisions.

9

References

1. Breu K, Hemingway CJ, Strathern M, Bridger D. Workforce Agility: The New Employee Strategy for the Knowledge Economy. J Inf Technol. 2002 Mar;17(1):21–31.
2. Fink L, Neumann S. Gaining Agility through IT Personnel Capabilities: The Mediating Role of IT Infrastructure Capabilities. J Assoc Inf Syst. 2007 Aug;8(8):440–62.
3. Lu Y, Ramamurthy KR. Understanding the Link Between Information Technology Capability and Organizational Agility: An Empirical Examination. MIS Q. 2011;35(4):931–54.
4. Tallon PP, Pinsonneault A. Competing Perspectives on the Link Between Strategic Information Technology Alignment and Organizational Agility: Insights from a Mediation Model. MIS Q. 2011;35(2):463.
5. Roberts N, Grover V. Leveraging Information Technology Infrastructure to Facilitate a Firm’s Customer Agility and Competitive Activity: An Empirical Investigation. J Manag Inf Syst. 2012 Apr 8;28(4):231–70.
6. Chakravarty A, Grewal R, Sambamurthy V. Information Technology Competencies, Organizational Agility, and Firm Performance: Enabling and Facilitating Roles. Inf Syst Res. 2013 Dec;24(4):976–97.
7. Liu H, Ke W, Wei KK, Hua Z. The impact of IT capabilities on firm performance: The mediating roles of absorptive capacity and supply chain agility. Decis Support Syst. 2013 Feb;54(3):1452–62.
8. Lee O-K (Daniel), Sambamurthy V, Lim KH, Wei KK. How Does IT Ambidexterity Impact Organizational Agility? Inf Syst Res. 2015 Jun;26(2):398–417.
9. Lee O-K (Daniel), Xu P, Kuilboer J-P, Ashrafi N. Idiosyncratic values of IT-enabled agility at operation and strategic levels. Commun Assoc Inf Syst. 2016;39(1):242–66.
10. Chen Y, Wang Y, Nevo S, Benitez J, Kou G. Improving Strategic Flexibility with Information Technologies: Insights for Firm Performance in an Emerging Economy. J Inf Technol. 2017 Mar;32(1):10–25.
11. Ghasemaghaei M, Hassanein K, Turel O. Increasing firm agility through the use of data analytics: The role of fit. Decis Support Syst. 2017 Sep;101:95–105.
12. Mikalef P, Pateli A. Information technology-enabled dynamic capabilities and their indirect effect on competitive performance: Findings from PLS-SEM and fsQCA. J Bus Res. 2017 Jan;70:1–16.
13. Ravichandran T. Exploring the relationships between IT competence, innovation capacity and organizational agility. J Strateg Inf Syst. 2018 Mar;27(1):22–42.
14. Queiroz M, Tallon PP, Sharma R, Coltman T. The role of IT application orchestration capability in improving agility and performance. J Strateg Inf Syst. 2018 Mar;27(1):4–21.
15. Tsou H-T, Cheng CCJ. How to enhance IT B2B service innovation? An integrated view of organizational mechanisms. J Bus Ind Mark. 2018 Aug 6;33(7):984–1000.
16. Ashrafi A, Zare Ravasan A, Trkman P, Afshari S. The role of business analytics capabilities in bolstering firms’ agility and performance. Int J Inf Manage. 2019 Aug;47:1–15.
