# Supplementary material for: A meta-analysis on the effects of IT capability toward agility and performance: New directions for information systems research
Source: PLoS One. 2022 Oct 27;17(10):e0268761. doi: 10.1371/journal.pone.0268761 (PMC9612477; doi:10.1371/journal.pone.0268761)
Supplement: S2 Table — (PDF) [file pone.0268761.s003.pdf]

A meta-analysis on the effects of IT capability toward agility and performance: New directions for information systems research

S2 - Examples of studies on the relationship between IT capability and performance

Table S2 gives an overview of different reasons found in the literature for the effect of reactive and proactive IT capability toward organizational performance. The table presents exemplary studies in support of our conceptualization of reactive and proactive IT capability and their varying effects toward performance.

Table S2 – Overview of exemplary studies, their concepts and reasoning for the effects of reactive and proactive IT capability toward performance

Author (Year)	ITC Concept	Reasoning for ITCR-OP relationship	Reasoning for ITCP-OP relationship
**Bharadwaj, 2000** [1]	**ITCR:** IT infrastructure, human IT skills, intangible IT benefits	Organizations differentiate themselves through effective combination of IT resources that are complex and difficult to imitate.	
**Wade and Hulland 2004** [2]	**ITCR:** Inside-out IS capability<br>**ITCP:** Outside-in IS capability, spanning IS capability	Inside-out IS capabilities, such as infrastructure, technical skills, development, and cost-efficient operations improve sustainable advantage through their imitability, substitutability, and mobility characteristics.	Outside-in IS capabilities, such as external relationship management and market responsiveness, and spanning IS capabilities, such as IS-business partnerships and IS management/planning, improve advantage creation through their value, rarity, and appropriability characteristics.
**Bhatt and Grover 2005** [3]	**ITCR:** IT infrastructure<br>**ITCP:** IT business experience, relationship infrastructure	IT infrastructure represents an effective source of value through information sharing across functions, innovation, exploitation of business opportunities, and the flexibility to respond to changes in business strategy.	IT groups' understanding of the business and a positive relationship between IT and business cannot easily be replicated by competitors. IT groups with superior business strategy, competition,

			and opportunities can continue to leverage these advantages based on their absorptive capability.
**Ravichandran and Lertwongsatien 2005** [4]	**ITCR:** IS planning, IS development, IS support maturity, IS operations	Strong functional capabilities in the IS department are required to use IT to develop and enhance an organization's core competencies, providing a competitive advantage.	
**Karimi et al. 2007** [5]	**ITCR:** ERP capability	ERP capability enables cost-effective IS operations and market responsiveness, leading to cost and cycle time reduction, as well as quality, productivity, and customer service improvements.	
**Aral and Weill 2007** [6]	**ITCR:** IT skills, IT use, digital transactions, internet architecture	Systems of IT practices and competencies enable greater business value generation while reducing internal and external integration costs.	
**Bharadwaj et al. 2007** [7]	**ITCR:** Integrated IS capability	Integrated IS capability provides visibility to relevant customer, production, order, and market data, facilitates process integration, and provides seamless and consistent access to data. Improved visibility and information flow, centralized services, integrated and standardized activities reduce errors and delays in order processing.	
**Zhang et al. 2008** [8]	**ITCR:** IT management<br>**ITCP:** IT business partnerships	IT management capability helps to coordinate multifaceted activities that are needed for successful IT implementations, making the organizations more successful.	IT business partnerships increase trust between IT groups and business units by sharing risk and responsibility of IT applications, which has positive performance implications.
**Stoel and Muhanna 2009** [9]	**ITCR:** Internally-focused IT capabilities<br>**ITCP:** Externally-focused IT capabilities	Internally focused IT capabilities provide reliable services and products and minimize overhead costs.	Externally focused IT capabilities help to rapidly sense and respond to market changes and shifts in customer and supplier needs, increasing organizational performance.
**Wang et al. 2012** [10]	**ITCR:** IT support for core competencies, IT support for competitive strategies, IT capabilities	IT is used to support the planning and implementation of competitive strategies as well as to enhance and develop competencies. Strong IT capabilities can select and prioritize IS portfolios, ensure appropriate IT investment patterns, and align IT strategies with business strategies.	
**Chakravarty et al. 2013** [11]	**ITCR:** IT competencies	Increased IT competencies increase the business value derived from business functions, ensuring timely information availability. Information processing enables information utilization, which facilitates firm performance.	
**Chen et al. 2014** [12]	**ITCR:** IT capability	IT capability improves business processes by infusing them with swiftness, robustness, and flexibility, indicators for business process agility, which in turn helps organizations to improve different performance measures.	

**Lee et al. 2016** [13]	**ITCP:** IT-based knowledge management		IT-based knowledge management enables organizational units to share their capabilities and know-how to achieve better performance.
**Chen et al. 2017** [14]	**ITCR:** IT support for functionality-related competency<br>**ITCP:** IT support for market-access competency	IT support for functionality-related competency increases strategic flexibility to refocus resources and improve performance.	IT support for market-access competency improves strategic flexibility to recalibrate strategy for performance gains.
**Mikalef and Pateli 2017** [15]	**ITCP:** IT-enabled sensing capability		IT-enabled sensing capability improves capitalizing on the market by new or improved value propositions through focused deployment.
**Queiroz et al. 2018** [16]	**ITCR:** IT application orchestration capability	Building new combinations of IT resources and continuously updating portfolio of IT applications has positive performance implications.	

References

1. Bharadwaj AS. A Resource-Based Perspective on Information Technology Capability and Firm Performance: An Empirical Investigation. MIS Q. 2000 Mar;24(1):169.
2. Wade, Hulland. Review: The Resource-Based View and Information Systems Research: Review, Extension, and Suggestions for Future Research. MIS Q. 2004;28(1):107.
3. Bhatt GD, Grover V. Types of Information Technology Capabilities and Their Role in Competitive Advantage: An Empirical Study. J Manag Inf Syst. 2005 Nov 8;22(2):253–77.
4. Ravichandran T, Lertwongsatien C. Effect of information systems resources and capabilities on firm performance: A resource-based perspective. J Manag Inf Syst. 2005 Apr 8;21(4):237–76.
5. Karimi J, Somers TM, Bhattacherjee A. The Role of Information Systems Resources in ERP Capability Building and Business Process Outcomes. J Manag Inf Syst. 2007 Oct 8;24(2):221–60.
6. Aral S, Weill P. IT Assets, Organizational Capabilities, and Firm Performance: How Resource Allocations and Organizational Differences Explain Performance Variation. Organ Sci. 2007 Oct;18(5):763–80.
7. Bharadwaj S, Bharadwaj A, Bendoly E. The performance effects of complementarities between information systems, marketing, manufacturing, and supply chain processes. Inf Syst Res. 2007;18(4):437–53.
8. Zhang M, Sarker S, Sarker S. Unpacking the effect of IT capability on the performance of export-focused SMEs: A report from China. Inf Syst J. 2008;18(4):357–80.
9. Stoel MD, Muhanna WA. IT capabilities and firm performance: A contingency analysis of the role of industry and IT capability type. Inf Manag. 2009;46(3):181–9.
10. Wang N, Liang H, Zhong W, Xue Y, Xiao J. Resource Structuring or Capability Building? An Empirical Study of the Business Value of Information Technology. J Manag Inf Syst. 2012 Oct 8;29(2):325–67.
11. Chakravarty A, Grewal R, Sambamurthy V. Information Technology Competencies, Organizational Agility, and Firm Performance: Enabling and Facilitating Roles. Inf Syst Res. 2013 Dec;24(4):976–97.
12. Chen Y, Wang Y, Nevo S, Jin J, Wang L, Chow WS. IT capability and organizational performance: The roles of business process agility and environmental factors. Eur J Inf Syst. 2014 May 19;23(3):326–42.
13. Lee O-K (Daniel), Xu P, Kuilboer J-P, Ashrafi N. Idiosyncratic values of IT-enabled agility at operation and strategic levels. Commun Assoc Inf Syst. 2016;39(1):242–66.
14. Chen Y, Wang Y, Nevo S, Benitez J, Kou G. Improving Strategic Flexibility with Information Technologies: Insights for Firm Performance in an Emerging Economy. J Inf Technol. 2017 Mar;32(1):10–25.
15. Mikalef P, Pateli A. Information technology-enabled dynamic capabilities and their indirect effect on competitive performance: Findings from PLS-SEM and fsQCA. J Bus Res. 2017 Jan;70:1–16.
16. Queiroz M, Tallon PP, Sharma R, Coltman T. The role of IT application orchestration capability in improving agility and performance. J Strateg Inf Syst. 2018 Mar;27(1):4–21.
