# Supplementary material for: A meta-analysis on the effects of IT capability toward agility and performance: New directions for information systems research
Source: PLoS One. 2022 Oct 27;17(10):e0268761. doi: 10.1371/journal.pone.0268761 (PMC9612477; doi:10.1371/journal.pone.0268761)
Supplement: S3 Table — (PDF) [file pone.0268761.s004.pdf]

A meta-analysis on the effects of IT capability toward agility and performance: New directions for information systems research

S4 - List of included studies, sample sizes and, correlations

Table S4 – Overview of primary studies and correlations included in the meta-analysis

Dataset	Reference	Outlet	Sample Size	OA-ITCR	OA-ITCP	OP-ITCR	OP-ITCP
**Akter et al. 2016**	[1]	IJPE	152				0.39
**Albesher 2014**	[2]	Thesis	186			0.15	
**Aral and Weill 2007**	[3]	OrgS	147			0.02	
**Ashrafi et al. 2019**	[4]	IJIM	154		0.34		0.44
**Benitez et al. 2018**	[5]	I&M	203	0.23		0.17	
**Bhatt et al. 2010**	[6]	I&M	105	0.64		0.61	
**Breu et al. 2002**	[7]	JIT	515	0.26			
**Brusset 2016**	[8]	IJPE	171	0.52			
**Cao et al. 2019**	[9]	IJIM	633			0.58	
**Chakravarty et al. 2013**	[10]	ISR	109	0.25		0.22	
**Chau Chung Kei 2011**	[11]	Thesis	111	0.56	0.38		
**Chen 2012**	[12]	Thesis	214	0.35		0.26	
**Chen et al. 2015**	[13]	I&M	138			0.28	0.24
**Chen et al. 2017**	[14]	JIT	148	0.19		0.24	
**DeGroote and Marx 2013**	[15]	IJIM	193			0.50	0.47
**Eichman 2013**	[16]	Thesis	115				0.34
**Fink and Neumann 2007**	[17]	JAIS	293	0.54	0.60		
**Ghasemaghaei et al. 2017**	[18]	DSS	215	0.56			
**Hazen et al. 2017**	[19]	IJPE	190	0.74	0.67	0.27	0.31
**Heim and Peng 2010**	[20]	JOM	238			0.06	
**Jiang and Zhao 2015**	[21]	IJNVO	184	0.52	0.58	0.42	0.44
**Karahanna and Preston 2013**	[22]	JMIS	81				0.30
**Kharabe et al. 2013**	[23]	ICIS	215	0.47	0.43		
**Kim and Chai 2017**	[24]	IJPE	272	0.02			
**Kim et al. 2011**	[25]	JAIS	243				0.33
**Lee et al. 2015**	[26]	ISR	178	0.20	0.33		
**Lee et al. 2016**	[27]	CAIS	195	0.32	0.29	0.20	0.24
**Liang et al. 2017**	[28]	ISR	429		0.10		
**Liu et al. 2009**	[29]	AMCIS	286	0.54		0.35	
**Liu et al. 2013**	[30]	DSS	286			0.42	
**Lowry and Wilson 2016**	[31]	JSIS	400			0.79	0.80
**Lu and Ramamurthy 2011**	[32]	MISQ	128	0.26	0.39		
**Mao et al. 2015**	[33]	ID	123		0.51		
**Mikalef and Pateli 2017**	[34]	JBR	274	0.48	0.55	0.42	0.45
**Mikalef et al. 2020**	[35]	EJIS	322			0.43	0.49
**Mithas et al. 2011**	[36]	MISQ	160			0.79	

**Pavlou and El Sawy 2010**	[37]	ISR	507	0.37		0.22	
**Qrunfleh and Tarafdar 2014**	[38]	IJPE	205	0.15	0.18	0.18	0.17
**Queiroz et al. 2018**	[39]	JSIS	120	0.44		0.23	
**Rai and Tang 2010**	[40]	ISR	318	0.39		0.36	
**Ramakrishnan et al. 2020**	[41]	CAIS	154	0.49		0.55	
**Raschke 2010**	[42]	IJAIS	273	0.31		0.35	
**Ravichandran 2018**	[43]	JSIS	129	0.39	0.41	0.23	0.33
**Rivard et al. 2006**	[44]	JSIS	96			0.40	0.44
**Roberts and Grover 2012**	[45]	JMIS	108	0.29	0.08	0.23	0.29
**Sanders 2007**	[46]	JOM	245			0.30	
**Saraf et al. 2007**	[47]	ISR	63			0.27	
**Stoel and Muhanna 2009**	[48]	I&M	686			-0.05	0.04
**Swafford et al. 2006**	[49]	IJO&PM	135	0.09	0.15	0.08	0.12
**Tallon 2008**	[50]	ITM	241	0.48	0.50		
**Tallon and Pinsonneault 2011**	[51]	MISQ	241	0.46		0.08	
**Torres et al. 2018**	[52]	I&M	266	0.45		0.30	
**Tsou and Cheng 2018**	[53]	JB&IM	170	0.35	0.30	0.36	0.32
**Wamba et al. 2017**	[54]	JBR	297				0.35
**Wang et al. 2012**	[55]	JMIS	296			0.22	0.23
**Wang et al. 2013**	[56]	CAIS	65			0.42	
**Wang et al. 2015**	[57]	JBR	235			0.28	
**Wei et al. 2020**	[58]	DS	215			0.30	0.24
**Wu and Li 2008**	[59]	IEEE AMIGE	215	0.44	0.53	0.54	0.52
**Youn et al. 2014**	[60]	IJIM	74		0.14		-0.04
**Zhou et al. 2018**	[61]	I&M	119	0.58	0.46		
**Zhu 2004**	[62]	JMIS	114			0.21	

NOTE: Outlet acronyms: AMCIS = Americas Conference on Information Systems; CAIS = Communications of the Association for Information Systems; DS = Decision Sciences; DSS = Decision Support System; EJIS = European Journal of Information Systems; I&M = Information & Management; ICIS = International Conference on Information Systems; ID = Information Development; IJAIS = International Journal of Accounting Information Systems; IJIM = International Journal of Information Management; IJNVO = International Journal of Networking and Virtual Organisations; IJO&PM = International Journal of Operations & Production Management; IJPE = International Journal of Production Economics; ISR = Information Systems Research; ITM = Information Technology Management; JAIS = Journal of the Association for Information Systems; JB&IM = Journal of Business & Industrial Marketing; JBR = Journal of Business Research; JIT = Journal of Information Technology; JM&O = Journal of Management & Organization; JMIS = Journal of Management Information Systems; JOM = Journal of Operations Management; JSIS = Journal of Strategic Information Systems; MISQ = Management Information Systems Quarterly; OrgS = Organization Science; PACIS = Pacific Asia Conference on Information Systems.

References

1. Akter S, Wamba SF, Gunasekaran A, Dubey R, Childe SJ. How to improve firm performance using big data analytics capability and business strategy alignment? Int J Prod Econ. 2016 Dec;182:113–31.
2. Albesher A. Synergies of Firms' Innovation Dynamic Capabilities and Information Technology. Brunel University; 2014.
3. Aral S, Weill P. IT Assets, Organizational Capabilities, and Firm Performance: How Resource Allocations and Organizational Differences Explain Performance Variation. Organ Sci. 2007 Oct;18(5):763–80.
4. Ashrafi A, Zare Ravasan A, Trkman P, Afshari S. The role of business analytics capabilities in bolstering firms' agility and performance. Int J Inf Manage. 2019 Aug;47:1–15.
5. Benitez J, Llorens J, Braojos J. How information technology influences opportunity exploration and exploitation firm's capabilities. Inf Manag. 2018;55(4):508–23.
6. Bhatt G, Emdad A, Roberts N, Grover V. Building and leveraging information in dynamic environments: The role of IT infrastructure flexibility as enabler of organizational responsiveness and competitive advantage. Inf Manag. 2010 Dec;47(7–8):341–9.
7. Breu K, Hemingway CJ, Strathern M, Bridger D. Workforce Agility: The New Employee Strategy for the Knowledge Economy. J Inf Technol. 2002 Mar;17(1):21–31.
8. Brusset X. Does supply chain visibility enhance agility? Int J Prod Econ. 2016 Jan;171:46–59.
9. Cao G, Duan Y, Cadden T. The link between information processing capability and competitive advantage mediated through decision-making effectiveness. Int J Inf Manage. 2019;44:121–31.
10. Chakravarty A, Grewal R, Sambamurthy V. Information Technology Competencies, Organizational Agility, and Firm Performance: Enabling and Facilitating Roles. Inf Syst Res. 2013 Dec;24(4):976–97.
11. Chau Chung Kei D. IT Governance and Agility - Organizational Information Processing Perspective. Honk Kong Polytechnic University; 2011.
12. Chen X. Impact of Business Intelligence and IT Infrastructure Flexibility on Competitive Advantage: An Organizational Agility Perspective. University of Nebraska; 2012.
13. Chen Y, Wang Y, Nevo S, Benitez-Amado J, Kou G. IT capabilities and product innovation performance: The roles of corporate entrepreneurship and competitive intensity. Inf Manag. 2015;52(6):643–57.
14. Chen Y, Wang Y, Nevo S, Benitez J, Kou G. Improving Strategic Flexibility with Information Technologies: Insights for Firm Performance in an Emerging Economy. J Inf Technol. 2017 Mar;32(1):10–25.
15. DeGroote SE, Marx TG. The impact of IT on supply chain agility and firm performance: An empirical investigation. Int J Inf Manage. 2013 Dec;33(6):909–16.
16. Eichman BW. An examination of the correlative effects of it outsourcing with it agility, it strategic alignment and it effectiveness. ProQuest Dissertations and Theses. 2013.
17. Fink L, Neumann S. Gaining Agility through IT Personnel Capabilities: The Mediating Role of IT Infrastructure Capabilities. J Assoc Inf Syst. 2007 Aug;8(8):440–62.
18. Ghasemaghaei M, Hassanein K, Turel O. Increasing firm agility through the use of data analytics: The role of fit. Decis Support Syst. 2017 Sep;101:95–105.
19. Hazen BT, Bradley R V., Bell JE, In J, Byrd TA. Enterprise architecture: A competence-based approach to achieving agility and firm performance. Int J Prod Econ. 2017 Nov;193(August):566–77.
20. Heim GR, Peng DX. The impact of information technology use on plant structure, practices, and performance: An exploratory study. J Oper Manag. 2010 Mar;28(2):144–62.
21. Jiang Y, Zhao J. The creative process of business value of IT in dynamic environment: the mediating effect of firm agility. Int J Netw Virtual Organ. 2015;15(2/3):200.
22. Karahanna E, Preston D. The effect of social capital of the relationship between the cio and top

management team on firm performance. J Manag Inf Syst. 2013;30(1):15–56.

23. Kharabe A, Lyytinen K, Grover V. Do Organizational Competencies Influence How Enterprise Systems Foster Organizational Agility? In: Thirty Fourth International Conference on Information Systems. Milan, IT: AISeL; 2013. p. 1–18.
24. Kim M, Chai S. The impact of supplier innovativeness, information sharing and strategic sourcing on improving supply chain agility: Global supply chain perspective. Int J Prod Econ. 2017 May;187(May 2016):42–52.
25. Kim G, Shin B, Kim KK, Lee HG. IT Capabilities, Process-Oriented Dynamic Capabilities, and Firm Financial Performance. J Assoc Inf Syst. 2011;12(7):487–517.
26. Lee O-K (Daniel), Sambamurthy V, Lim KH, Wei KK. How Does IT Ambidexterity Impact Organizational Agility? Inf Syst Res. 2015 Jun;26(2):398–417.
27. Lee O-K (Daniel), Xu P, Kuilboer J-P, Ashrafi N. Idiosyncratic values of IT-enabled agility at operation and strategic levels. Commun Assoc Inf Syst. 2016;39(1):242–66.
28. Liang H, Wang N, Xue Y, Ge S. Unraveling the Alignment Paradox: How Does Business—IT Alignment Shape Organizational Agility? Inf Syst Res. 2017 Dec;28(4):863–79.
29. Liu H, Ke W, Wei KK, Huang Q, Gu J, Chen H. From IT capabilities to supply chain performance: The mediating effects of supply chain agility and absorptive capacity. In: 15th Americas Conference on Information Systems 2009, AMCIS 2009. San Fransisco, CA, USA: AISeL; 2009. p. 1706–18.
30. Liu H, Ke W, Wei KK, Hua Z. The impact of IT capabilities on firm performance: The mediating roles of absorptive capacity and supply chain agility. Decis Support Syst. 2013 Feb;54(3):1452–62.
31. Lowry PB, Wilson D. Creating agile organizations through IT: The influence of internal IT service perceptions on IT service quality and IT agility. J Strateg Inf Syst. 2016 Oct;25(3):211–26.
32. Lu Y, Ramamurthy KR. Understanding the Link Between Information Technology Capability and Organizational Agility: An Empirical Examination. MIS Q. 2011;35(4):931–54.
33. Mao H, Liu S, Zhang J. How the effects of IT and knowledge capability on organizational agility are contingent on environmental uncertainty and information intensity. Inf Dev. 2015 Sep 4;31(4):358–82.
34. Mikalef P, Pateli A. Information technology-enabled dynamic capabilities and their indirect effect on competitive performance: Findings from PLS-SEM and fsQCA. J Bus Res. 2017 Jan;70:1–16.
35. Mikalef P, Pateli A, van de Wetering R. IT architecture flexibility and IT governance decentralisation as drivers of IT-enabled dynamic capabilities and competitive performance: The moderating effect of the external environment. Eur J Inf Syst. 2020 Aug 27;1–29.
36. Mithas, Ramasubbu, Sambamurthy. How Information Management Capability Influences Firm Performance. MIS Q. 2011;35(1):237.
37. Pavlou PA, El Sawy OA. The “Third Hand”: IT-Enabled Competitive Advantage in Turbulence Through Improvisational Capabilities. Inf Syst Res. 2010 Sep;21(3):443–71.
38. Qrunfleh S, Tarafdar M. Supply chain information systems strategy: Impacts on supply chain performance and firm performance. Int J Prod Econ. 2014 Jan;147(PART B):340–50.
39. Queiroz M, Tallon PP, Sharma R, Coltman T. The role of IT application orchestration capability in improving agility and performance. J Strateg Inf Syst. 2018 Mar;27(1):4–21.
40. Rai A, Tang X. Leveraging IT capabilities and competitive process capabilities for the management of interorganizational relationship portfolios. Inf Syst Res. 2010;21(3):516–42.
41. Ramakrishnan T, Khuntia J, Kathuria A, Saldanha TJV. An integrated model of business intelligence & analytics capabilities and organizational performance. Commun Assoc Inf Syst. 2020;46:722–50.
42. Raschke RL. Process-based view of agility: The value contribution of IT and the effects on process outcomes. Int J Account Inf Syst. 2010 Dec;11(4):297–313.
43. Ravichandran T. Exploring the relationships between IT competence, innovation capacity and organizational agility. J Strateg Inf Syst. 2018 Mar;27(1):22–42.
44. Rivard S, Raymond L, Verreault D. Resource-based view and competitive strategy: An integrated model of the contribution of information technology to firm performance. J Strateg Inf Syst. 2006;15(1):29–50.
45. Roberts N, Grover V. Leveraging Information Technology Infrastructure to Facilitate a Firm’s

Customer Agility and Competitive Activity: An Empirical Investigation. J Manag Inf Syst. 2012 Apr 8;28(4):231–70.
46. Sanders NR. An empirical study of the impact of e-business technologies on organizational collaboration and performance. J Oper Manag. 2007;25(6):1332–47.
47. Saraf N, Langdon CS, Gosain S. IS application capabilities and relational value in interfirm partnerships. Inf Syst Res. 2007;18(3):320–39.
48. Stoel MD, Muhanna WA. IT capabilities and firm performance: A contingency analysis of the role of industry and IT capability type. Inf Manag. 2009;46(3):181–9.
49. Swafford PM, Ghosh S, Murthy NN. A framework for assessing value chain agility. Int J Oper Prod Manag. 2006 Feb 11;26(2):118–40.
50. Tallon PP. Inside the adaptive enterprise: an information technology capabilities perspective on business process agility. Inf Technol Manag. 2008 Mar 4;9(1):21–36.
51. Tallon PP, Pinsonneault A. Competing Perspectives on the Link Between Strategic Information Technology Alignment and Organizational Agility: Insights from a Mediation Model. MIS Q. 2011;35(2):463.
52. Torres R, Sidorova A, Jones MC. Enabling firm performance through business intelligence and analytics: A dynamic capabilities perspective. Inf Manag. 2018;55(7):822–39.
53. Tsou H-T, Cheng CCJ. How to enhance IT B2B service innovation? An integrated view of organizational mechanisms. J Bus Ind Mark. 2018 Aug 6;33(7):984–1000.
54. Wamba SF, Gunasekaran A, Akter S, Ren SJ, Dubey R, Childe SJ. Big data analytics and firm performance: Effects of dynamic capabilities. J Bus Res. 2017 Jan;70(2):356–65.
55. Wang N, Liang H, Zhong W, Xue Y, Xiao J. Resource Structuring or Capability Building? An Empirical Study of the Business Value of Information Technology. J Manag Inf Syst. 2012 Oct 8;29(2):325–67.
56. Wang Y, Chen Y, Nevo S, Jin J, Tang G, Chow WS. IT capabilities and innovation performance: The mediating role of market orientation. Commun Assoc Inf Syst. 2013;33(1):129–48.
57. Wang G, Dou W, Zhu W, Zhou N. The effects of firm capabilities on external collaboration and performance: The moderating role of market turbulence. J Bus Res. 2015;68(9):1928–36.
58. Wei S, Ke W, Liu H, Wei KK. Supply Chain Information Integration and Firm Performance: Are Explorative and Exploitative IT Capabilities Complementary or Substitutive? In: Decision Sciences. 2020. p. 464–99.
59. Wu J, Li R. Modeling the Relationship between IT Capability, Uncertainty and Organizational Performance: An Empirical Study. In: 2008 IEEE Symposium on Advanced Management of Information for Globalized Enterprises (AMIGE). IEEE; 2008. p. 1–5.
60. Youn SH, Yang MG (Mark), Kim JH, Hong P. Supply chain information capabilities and performance outcomes: An empirical study of Korean steel suppliers. Int J Inf Manage. 2014 Jun;34(3):369–80.
61. Zhou J, Bi G, Liu H, Fang Y, Hua Z. Understanding employee competence, operational IS alignment, and organizational agility – An ambidexterity perspective. Inf Manag. 2018 Sep;55(6):695–708.
62. Zhu K. The complementarity of information technology infrastructure and E-commerce capability: A Resource-based assessment of their business value. J Manag Inf Syst. 2004;21(1):167–202.
