# Supplementary material for: A meta-analysis on the effects of IT capability toward agility and performance: New directions for information systems research
Source: PLoS One. 2022 Oct 27;17(10):e0268761. doi: 10.1371/journal.pone.0268761 (PMC9612477; doi:10.1371/journal.pone.0268761)
Supplement: S1 File — (PDF) [file pone.0268761.s005.pdf]

A meta-analysis on the effects of IT capability toward agility and performance: New directions for information systems research

S3 - Details for the computerized search

In the following, we describe our computerized search strategy in more details. We conducted a comprehensive computerized search along the Technology-Organization-Environment-Framework [1]. The technology dimension helped us to include relevant studies considering IT antecedents; the organization dimension focused on studies from the organizational literature; the environment dimensions identified influential studies considering environmental factors. We customized the set of databases and defined different search parameters for each topical search (see Table C1). For example, for the technology dimensions we include databases from the technical domain, such as IEEE Xplore and ACM Digital Library. Given much research on IT capability comes from information systems researchers, we made sure to include information systems outlet in each topical search. We searched more comprehensively and included different academic publications, such as journals and conferences, in our search.

Table S3 – Overview of databases, search strings and inclusion criteria for computerized search

	Technology	Organization	Environment
**Database**	EBSCO, IEEE Xplore, ProQuest, ACM Digital Library, Science Direct and AISeL	Journal: EBSCO Academic Search Complete, EBSCO Business Source Complete, ProQuest, and ScienceDirect<br>Conferences: ICIS, ECIS, PACIS, AMCIS, HICSS	ACM Digital Library, AISeL, EBSCO Business Source Complete, ProQuest ABI Inform, ScienceDirect and Scopus.
**Search String**	Agility AND (Performance OR Efficiency) AND (Organi* OR Firm OR Business) AND (Information System* OR Enterprise System* OR Technology)	Journal: Agil* AND (performance OR productivity OR efficiency OR effectiveness OR "return on investment" OR sales OR satisfaction OR profitability OR growth OR success) AND (organi*ation OR firm OR enterprise OR company OR corporate OR business)<br>Conference: Agil* AND (organi*ation OR firm OR enterprise OR company OR corporate OR business)	(Agility OR Agile) AND (Performance) AND (Environment OR Environmental OR Industry OR Government OR Governmental OR Market OR Regulation)
**Inclusion and Exclusion Criteria**	• Publication is written in English<br>• Publication reports on completed empirical research<br>• Publication is thematically relevant, that is, the article focuses on organizational phenomenon related agility and performance<br>• Publication is not qualitative research or a literature review		
NOTE: ICIS = International Conference on Information Systems, ECIS = European Conference on Information Systems, PACIS = Pacific Asia Conference on Information Systems, AMCIS = Americas Conference on Information Systems, HICSS = Hawaii International Conference on System Sciences; AISeL = Association for Information Systems Electronic Library.			

References

1. Depietro R, Wiarda E, Fleischer M. The context for change: Organization, technology and environment. In: Tornatzky, L.G. and Fleischer M, editor. The processes of technological innovation. Lexington, MA, USA: Lexington Books; 1990. p. 151–75.
